# Wanted, a Constructive Policy

**Published:** 1908-08-08

**Authors:** 


### Nursing Administration.

**Correspondence and Queries** for this section should be sent to the Editor of The Hospital, 28 Southampton Street, Strand, London, and marked "Nursing Administration."

### WANTED, A CONSTRUCTIVE POLICY.

The registration of nurses is a subject which is in the mind of every matron, and it is hardly too much to say that each one has her own theories of what can, as well as what cannot, be wisely attempted in this direction. It would be the gravest possible mistake to conclude that the interest taken in this important question by the heads of training schools is to be measured by the scanty list of names attached to the petition recently presented to the Government in favour of legislation. But are there not signs that the moment for inaction is passing away, and that opposition to measures deemed by the majority highly inexpedient in the interests of nursing generally should now yield to a definite constructive policy? The rank and file of nurses generally are uneasily anxious for a more generally acknowledged status. Abuses flourish in the absence of precautions taken against them. The control of nursing agencies and of nursing homes is lodged in unsuitable hands, and dominated solely by commercial motives. Nursing education is what each individual matron chooses to make it. And withal the Government shows a laudable disposition to grant facilities for legislation. All that is needed is for the real controlling forces in nursing, the nurse-training schools, to draw together and formulate a policy which should constitute a working basis for agreement. Supported as such a policy would be by thousands of enthusiastic adherents, the matter could thus be settled by consensus of opinion on lines admitting of natural growth and ensuring widespread reform. One point appears to be essential to the success of any legislation for nurses. Experience, not merely in this country, has shown that nurses as a body cannot be counted on to respond universally to any call, however insistent, to band themselves together for the common good. The forces which draw them together are less powerful than the forces which keep them apart. However overwhelming might be the advantages of being enrolled in an organised body, there would still be a large proportion who would remain outsiders—some because they did not see what immediate advantage they stood to gain, some because they belonged to an opposition camp, some from sheer laziness. If nurses are ever to become a strong, well-organised body, graded in classes, or, as many think would be better, officially credited with such varied, though unclassified, qualifications as they might possess, it will never be through their own initiative. The onus of enrolling them under whatever code of regulations may be decided on must rest on the schools in which they are trained, and the process of certification and enrolment must be so closely connected that every nurse, on the completion of her training, if fit to be certificated at all, must pass at once and automatically into the great organised body of trained nurses amenable to one code and with a recognised status. Thus and thus only after the lapse of a few years could the unity which all desire be attained. So long as there is a loophole for the existence of two classes of trained nurses, one belonging to the central body, whether by virtue of registration or examination; and the other, equally well trained, electing to stand outside, so long will a central examination remain an empty counsel of perfection, and the abuses of the nursing world, unaffected by the organisation of a certain proportion of nurses, including many not the best, will flow on unchecked.

The countries in which registration has been a distinct success are countries in which hospitals are under State control, the certifying body and the registering body being in complete accord. Where this is the case, nothing is left to the choice of the individual nurse. As she completes her training she is drafted into the ranks of trained nurses, and unless she can prove her membership of this body, she is definitely disqualified from following her avocation, except under the gravest disadvantages. Under the voluntary hospital system it is impossible to obtain the same results in the same way. To attempt to force outside regulations upon independent institutions which control both training and employment, and are hostile towards the central body is to court failure. If the governing body which enrols is to be in complete accord with the certifying bodies which train, is it not clear that the nurse-training schools must themselves draw together and formulate a working scheme which they could consent loyally to support? In our judgment there are many signs that agreement is possible, that ideas are not lacking, and that the time for inaction is over. The difficulties in the way of a dignified settlement of this important question would be enormously multiplied by the passing of a measure to which the main training centres were with good reason avowedly antagonistic. A central board without public confidence, regulations made which no one could be compelled to obey, a hall-mark which would impress only the non-employer, and after a year or two of simmering agitation, a perception that everything was going on much as before, this is what registration appears to promise as now before the public. Yet, if once established on futile or wrong-headed lines, registration would bar the way to more definite progress and reform. Parliament is never lightly disposed to readjust measures undertaken in the interests of a special class, and what is done will be practically all that is attempted, at any rate in this generation. If we are not to see one more abortive and costly experiment undertaken in the vain belief that nurses can be induced to act spontaneously together for their own and the common good, it is clear that the required spontaneous action must be taken by the training schools who are authorised to speak in the names of nurses generally. A conference of the right people, assembling with the determination to find a basis of agreement, would be able to prove that the supposed insurmountable difficulties are more in appearance than in reality.

